# Sharing-based social capital associated with harvest production and wealth in the Canadian Arctic

**DOI:** 10.1371/journal.pone.0193759

**Published:** 2018-03-12

**Authors:** Elspeth Ready

**Affiliations:** Department of Sociology, University of Nebraska-Lincoln, Lincoln, NE, United States of America; University of South Carolina, UNITED STATES

## Abstract

Social institutions that facilitate sharing and redistribution may help mitigate the impact of resource shocks. In the North American Arctic, traditional food sharing may direct food to those who need it and provide a form of natural insurance against temporal variability in hunting returns within households. Here, network properties that facilitate resource flow (network size, quality, and density) are examined in a country food sharing network comprising 109 Inuit households from a village in Nunavik (Canada), using regressions to investigate the relationships between these network measures and household socioeconomic attributes. The results show that although single women and elders have larger networks, the sharing network is not structured to prioritize sharing towards households with low food availability. Rather, much food sharing appears to be driven by reciprocity between high-harvest households, meaning that poor, low-harvest households tend to have less sharing-based social capital than more affluent, high-harvest households. This suggests that poor, low-harvest households may be more vulnerable to disruptions in the availability of country food.

## Introduction

In many communities worldwide, food access is not only determined by income, food prices, or agricultural yields but also by traditional rights to land or resources and by formal or informal social institutions. For example, strong sharing norms, particularly surrounding the sharing of food, were historically common among indigenous populations in the Arctic and subarctic. Today, sharing of traditional “country” foods, such as caribou and seal, remains an important component of food access in remote northern settlements throughout North America [1–10]. More broadly, food sharing is important in remote communities around the globe where people, often indigenous groups, depend on foraged food [11–14], and has also been documented as a strategy used to improve food access in rural food deserts in the United States [15].

Climate change will have important impacts on all dimensions of global food security, including food availability, stability, access, and utilization [16]. In the Arctic specifically, processes related to climate change, such as coastal erosion and reduced sea ice, are already having considerable impacts on local communities (e.g., [17, 18]). Modelling exercises based on both scientific research and Traditional Ecological Knowledge suggest that the availability of subsistence resources to harvesters in Alaska will decline over the next 30 years, primarily as a result of climate-related barriers to resource access (especially unsafe travel conditions) [19].

Given the ongoing impacts of climate change in the Arctic, many studies have focused on identifying how local communities might be negatively affected and what coping strategies or ‘adaptations’ may mitigate these negative effects (e.g., [20–24]). Studies of the potential effects of climate change on Canadian Inuit food systems have suggested that food sharing may buffer Inuit food systems from climate risks, because sharing channels country food to individuals who do not hunt and provides households with access to country food when certain hunters are unable to access the land [25–27]. The assumption that sharing provides insurance for hunters and support for the needy has long been an intuitively appealing functional explanation of food sharing among Inuit and other groups that rely on hunting for all or part of their subsistence, and variants of this explanation have long been espoused by subarctic and Arctic ethnographers (e.g., [28, 29]). However, climate change is only one part of a broad suite of challenges facing Inuit and other modern hunter-gatherers today, including population growth, health transitions, increased market integration and other changes that may also affect the dynamics of food sharing [14, 30].

While food sharing undeniably remains an integral component of sociality and identity in northern Canadian indigenous communities today [4, 9, 31], flows of food and sharing network structures have rarely been empirically investigated (although see [5, 10, 32] for exceptions). As such, the argument that food sharing prioritizes the needy and/or serves as insurance for hunters remains a largely untested assertion. Here, I examine the relationships between household wealth, harvest production, and food-sharing network structures in Kangiqsujuaq, Nunavik, Canada, in order to demonstrate how food sharing networks may complement or compensate for other forms of food access. In particular, I draw on the concept of network social capital to examine how the structure of social relations, as represented by sharing ties, may help households access resources.

### Sharing-based social capital

Social capital can help individuals and groups access resources and mobilize for collective action, and so the distribution of social capital among individuals and groups is important for understanding vulnerability and adaptive capacity in the face of environmental risks [33]. There are two common conceptualizations of social capital [34] (p. 9). The first defines social capital as “access to and use of resources embedded in social networks” (e.g., [35, 36]), while the second considers social capital as an emergent property of networks that enhances the “solidarity and reproduction of groups” (e.g., [37, 38]). These approaches differ in treating social capital as a private good, under the former definition, versus as a collective good, under the latter. By facilitating the distribution of resources or the flow of information, or by fostering group solidarity, social capital can enhance the ability of individuals or groups to cope with change (i.e., their adaptive capacity) [33].

Although sharing ties strengthen and may even create close social bonds between sharing partners, they fundamentally involve material flows. Consequently, I consider food sharing as a form of social capital that provides access to resources in a network [35, 39]. To examine how sharing-based social capital is distributed in Kangiqsujuaq, I therefore examine structures in the food-sharing network that can enhance a household’s ability to obtain country food from its connections, or ego-network.

**Fig 1:**
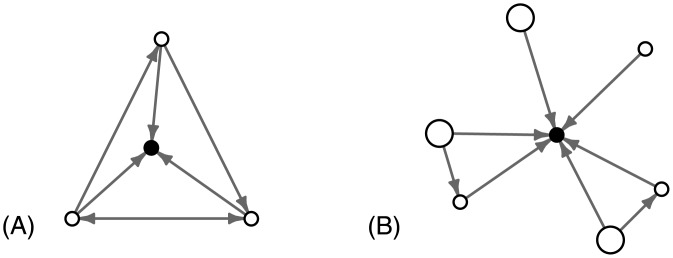
Illustrations of the household network properties associated with potential resource flow. The household in the center of network (B) has more incoming ties, more high quality ties (large nodes), and lower density than the household in the center of network (A).

Models of resource flow suggest three network characteristics that have the potential to enhance resource flow to households [40], illustrated in Fig 1. First, a large number of incoming sharing ties (high in-degree) are predicted to improve a household’s chances of receiving shares of food even if some links in the network are removed [41, 42]. Second, the compositional quality of a household’s sharing network is also important: ties to households that have more resources to share will be advantageous. Third, in-ties from uncorrelated sources might also be advantageous, because a household occupying a bridge between otherwise unconnected households would have access to more diverse (i.e., more independent) food sources [35, 41]. To decrease the correlation in resource availability, households should therefore be connected to households, or to groups of households, that are not connected to each other. This means that their ego-networks should have lower density for a given size, where density refers to the proportion of pairs of households in the network that are connected to each other [43]. I refer to these three network properties as sharing-based social capital.

Network effects, such as homophily, can increase social inequality, partially because they may result in differential access to social capital [39, 44]. The association of sharing-based social capital with other resources, including material or economic capital (such as income and hunting equipment) and embodied capital (in this context, hunting knowledge and skill [45]) is therefore important for understanding the role of food sharing in promoting well-being within households and in the settlement more broadly. Here, I consider two hypotheses for how sharing-based social capital might be distributed based on the interactions between different socioeconomic groups in Kangiqsujuaq. I focus specifically on the relationships between household wealth, household harvest production (which reflects access to the means of production, skill, and knowledge), and household sharing network structures.

First, sharing-based social capital might represent an alternative to other forms of resource access. This hypothesis reflects how many Kangiqsujuarmiut speak about the importance of sharing: those in need, particularly single mothers and the elderly, are prioritized. If this is consistently the case, households with lower economic or embodied capital should have greater sharing-based social capital than households with better resource access through non-social means. Therefore, we would expect the structures described above to surround households with less ability to access country food specifically (i.e., households with lower harvests) or food more generally (i.e., households with less wealth) than households with larger harvests or greater wealth. I refer to this as the “trickle-down” hypothesis. Through this mechanism, social capital could compensate for unequal access to other forms of capital.

Sharing-based social capital might also provide a form of insurance for harvesters against brief periods of bad luck, illness, or poor hunting conditions (e.g., [11, 46]), a hypothesis which is reflected in how some hunters in Kangiqsujuaq highlight the importance of sharing freshly-caught country food with other hunters. Under this hypothesis, sharing ties would be more likely to occur between high-production households, because high producers should target each other in order to maximize the probability that shares given will be reciprocated. In this case, sharing-based social capital should be positively correlated with other forms of capital in Kangiqsujuaq, particularly with higher harvests. I call this the “insurance” hypothesis.

I test these predictions in the Kangiqsujuaq sharing network data by examining the relationship between household food access and the properties of the ego-networks surrounding each household described above. In principle, these hypotheses are not mutually exclusive, as households might share for different reasons [47], and even for different reasons at different times. However, food security, wealth, investment in harvesting, and *giving* country food are positively correlated in the settlement [47, 48]. Consequently, the aggregate or emergent pattern of household-level food-sharing decisions is important for understanding the role of sharing in promoting food security across the settlement: do wealthy hunting households tend to prioritize those in need of food, or do they exchange food primarily with other similar households? If few hunters prioritize needy households, then sharing may not provide a very effective means of reducing the severity of food insecurity in the settlement. Yet at the same time, if too few hunters share with other hunters, sharing would not provide effective insurance.

The network dataset analyzed here, like many anthropological social network datasets (e.g., [30, 49]), lacks information on quantities exchanged. Instead, it consists of freelists of important sharing partners provided by respondents; it is intended to capture persistent relationships rather than ephemeral exchanges (see Data and Methods). Although an understanding of such relationships is important, the network is also therefore not a complete picture of flows of food in the community. Clearly, the amount of food given and received might vary considerably between sharing ties; some households may have a single tie that provides more food than several ties from other households. Unfortunately, it would be impossible to observe exchanges in the whole community, and Kangiqsujuarmiut generally do not pay close attention to amounts of food exchanged. Despite the lack of information on quantities exchanged, the analysis of network structures in established sharing relationships is nevertheless relevant to understanding how households might cope with changes to the distribution of resources in the network. A household with only one sharing tie, for example, would have fewer existing relationships to draw upon if something happened to their only connection (e.g., due to a hunter’s illness or injury) even if ordinarily, large amounts of food flowed along that tie. Similarly, a household with sharing ties only with low-producing households might have more seasonal constraints on country food access: in contrast to high-producing hunters who focus on different species throughout the year, many harvesters in low-production households only participate in fishing, berry picking, and mussel collecting during the spring and summer months. Consequently, network social capital, represented by the sharing network properties described above, is a useful means of investigating the potential vulnerability of households to social and environmental change.

### Research setting

Kangiqsujuaq is a small settlement located on the west coast of the Hudson Strait. Nearly all of the village’s roughly 750 permanent residents are Inuit. There are no roads to the settlement, so most imported goods are brought to the settlement by sealift during the summer months, while small quantities of perishable goods arrive by plane on a roughly weekly basis year-round. Data collected as part of this study indicate that 20% of Kangiqsujuarmiut had low food security and 21% had very low food security in 2013–2014, based on responses to a modified version of the HFSSM, a survey tool which primarily measures food access [48]. These frequencies are similar to those reported in previous studies in Nunavik and Nunavut [50–52]. Because of the need to import food and other goods, the cost of living in Nunavik is extremely high despite government subsidies on housing and many food items and commodities [53]. At the same time, un- and under-employment rates are very high: half of respondents to a 2008 health study in Nunavik did not have a full-time job [54]. This combination means that poverty and food insecurity are serious social problems in Kangiqsujuaq, as they are elsewhere in the Canadian Arctic [52].

The livelihoods of most households in Kangiqsujuaq involve wage labor, predominantly in the public sector, as well as participation in traditional harvesting activities. However, modern harvest participation is mediated by access to cash and equipment, as well as by the individuals’ interest in harvesting, and their hunting knowledge and ability [3, 5, 48, 55, 56]. As a result, harvest production levels vary considerably between households. Kangiqsujuarmiut hunt, fish, and gather a wide range of foods, including caribou, seal, beluga, mussels, ptarmigan, geese, and berries, although arctic char is the main country food staple. A 2002 Nunavik regional study reported that 12% of all calories consumed by Nunavimmiut came from country foods, representing 58% of total meat intake [57]. A 2004 24-hour recall study obtained a similar figure for women in Kangiqsujuaq (11%) [50].

Previous research suggests that over the past few decades, sharing practices in Inuit communities have changed as a result of the ever-increasing need for cash to support hunting activities [2, 3, 55, 58]. For example, in Ulukhaktok (Inuvialuit Settlement Region) households with greater involvement in the cash economy have become more isolated in the food sharing network [5]. Although sharing practices may be somewhat different than in the past, country food sharing in Kangiqsujuaq continues to take place in many different circumstances. This includes division of the catch among those who participated in a hunt, exchange of food among harvesters who encounter each other out on the land, gifts of both processed and unprocessed traditional foods between friends and family, as well as large-scale distributions of food by individual hunters who made a large catch or by numerous hunters after a community hunt. All sizes and types of country foods, from mussels to beluga, are shared. Many food sharing relationships are between kin; although by virtue of adoption practices, high fertility, and marriage, most individuals have large numbers of kin and choose which kin they prefer to interact with more or less frequently.

Sharing in Inuit communities, including Kangiqsujuaq, is not limited to country food. Commensalism, or sharing of meals with individuals outside of the immediate household, is also important; for example, young people will often eat with an older relative for lunch, people of all ages host meals for friends and family, and anyone present in the home at meal time will be served. Shared meals may or may not include country foods. Store-bought foods (crackers, cans of soup, bannock ingredients) are occasionally given, but usually only on request. Hunting equipment, spare parts, and tools are also shared, although many hunters in Kangiqsujuaq mentioned that to avoid damage and wear, they preferred not to share expensive equipment, such as snowmobiles, even with close relatives.

Selling country food to individuals is prohibited by the James Bay and Northern Quebec Agreement (the region’s land claim agreement). Although a small “black market” trade in country food exists, many Nunavimmiut disapprove of the practice [59]. Hunters are allowed to sell harvested foods to the local government-funded Hunter Support Program, which then makes the food freely available to the community, sometimes with preferential access for elders and needy families. However, the operation of the Hunter Support Program can be sporadic and unpredictable, and the food made available in the community freezer is sometimes of lower quality. Therefore, households’ own harvests and sharing relations (as described above), rather than markets or formal institutions, continue to be the primary means of access to country food.

## Data and methods

Data were collected in 2013–2014 in a household survey conducted as part of a 12-month ethnographic research project in Kangiqsujuaq. In total, 75% (110/145) of Inuit households in the community participated. The population sampled in the household survey shows the same age (Kolmogorov-Smirnov test: *D* = 0.0399, *p* = 0.7162) and sex (*χ*^2^ = 0.0498, *p* = 0.8234) distributions as the 2011 census data for Kangiqsujuaq [60]. In general, interviews were conducted jointly by the author and a local translator or research assistant. The study was approved by the Stanford IRB (Registration #IRB00000349, Protocol #26053), and all participants provided oral consent. 33% of households in the sample are headed by single women, all of whom support dependent children and/or adults.

The survey included questions about household demographics, employment, income, hunting participation, food sharing, and food security during the previous 12 months. I measure household access to food using two indicators: (1) household harvest production over a 12-month period, a measure of the country food directly available to the household, and (2) household wealth, which is associated with a household’s ability to purchase store food as well as supplies required for harvest production. I use the number of vehicles owned by the household, including all-terrain vehicles, snowmobiles, cars/trucks, fishing boats, and freighter canoes as a proxy of household material wealth. This measure is strongly correlated with household 12-month income, has no missing values, and also reflects wealth (and income management) over a longer term than recent income. The harvest data represent household catches of four important food species—ringed seal (*Phoca hispida*), beluga (*Delphinapterus leucas*), geese (*Branta canadensis* and *Chen caerulescens*), and caribou (*Rangifer tarandus*)—over a period of 12 months, converted to kilocalories and then binned into three categories: low-harvest households, which did not harvest any of the aforementioned species; super-households, which are households with harvests in the top 30% of harvest production levels [61, 62]; and mid-production households, with intermediate harvest levels. Arctic char harvests were not reported in the survey because respondents found it difficult to accurately estimate quantities of fish caught, even over relatively short time spans.

The food sharing network for the village was constructed using freelists of sharing ties provided by survey respondents. Respondents were asked to list who they shared country food with “most often,” including both households that they give to as well as households that give food to them. Respondents were allowed to name as many or as few partners as they wished, although they were prompted with the question “Anyone else?” until they responded “No.” The network analyzed here represents sharing of processed or unprocessed country food (the species listed above and others such as arctic char, mussels, and ptarmigan), and does not include sharing of food at meals or sharing of purchased food items. Households’ lists of sharing partners were then aggregated to create a network representing the sharing ties between all households that participated in the survey. This network is composed of 500 unique ties among 110 households, with a mean of 4.54 giving ties per household (range 0 to 32) and a mean of 4.54 incoming ties (range 0 to 16). Additional details on the construction and composition of the network have been published previously [47]. One “household” in the sample is not included in the analyses here due to special social and economic circumstances; however, sharing ties towards this household are included in the remaining households’ giving activities.

The three measures of network social capital described in the previous section (size, quality, and density) were calculated from each household’s local neighborhood (ego-network) in the overall network. I then conducted regressions to examine the relationships between these network properties and household attributes. In addition to the main variables of interest (i.e., household harvest production level and wealth), several variables that could be associated with household sharing-based social capital were considered in the analyses, including: (1) out-degree, meaning the number of outgoing sharing ties from a household, (2) household size, (3) age of the oldest household member, (4) whether the household was headed by a single female, (5) whether the household gave country food away over the local FM radio, and (6) the number of other households with siblings, parents, or children of household members. A control for the size of the household’s sharing network (in-degree, meaning the number of incoming sharing ties) was included in the regressions for network quality and density. The measure of network density used is the observed proportion of possible connections occurring between the households that provide food to the focal household, not including connections between households in the ego-network due to the focal household’s own sharing activities. Two households with no incoming sharing ties were assigned a network density of 1.0 (the highest possible value). The dataset used in the analyses is described in S1 Table and provided in S1 Data.

Analyses were conducted in R [63], using the igraph [64] and statnet [65] packages for the manipulation and analysis of the food sharing networks. Linear regressions were conducted in a Bayesian paradigm, via MCMC simulation [66, 67]. Models were run using the rjags [68] and coda [69] packages. Numeric variables were log-transformed (*log*(*x* + 1)) and centered around their means. All MCMC runs contained 10 000 samples taken after 10 000 burn-in samples. Trace and density plots of the MCMC samples, as well as Gelman and Rubin’s convergence diagnostic [70, 71] were examined for all models. Priors used for the models were diffuse, indicating a lack of strong prior information. For all *β* values, priors were normally distributed with a mean of zero and precision of 0.01. For the response variables, the priors were normally distributed around *μ* with *τ* distributed as Γ(0.01, 0.01).

## Results

**Fig 2:**
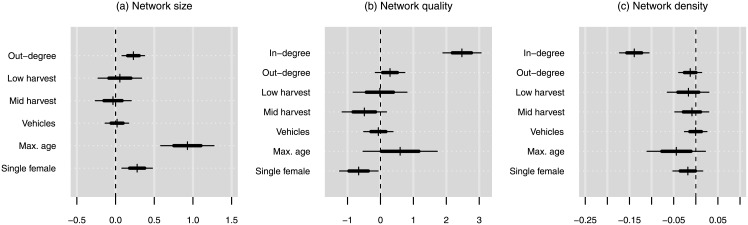
Posterior distributions of coefficients for selected predictor variables for the measures of network social capital. 68% and 95% quantiles of the distributions are shown by the thick and thin lines, respectively. The numeric variables, including the response variable, were log-transformed and centered. Low-harvest, mid-harvest, and single female are binary variables.

Regression results for the three proposed measures of network social capital are summarized in Fig 2, which shows the posterior distributions of the coefficients for several parameters of interest, including two categorical measures of harvest production (relative to the high-production reference category), the number of vehicles owned by the household, the age of the oldest household member, whether the household is headed by a single woman, the number of outward sharing ties that the household has (“out-degree”), and a control for incoming network size (“in-degree”) where appropriate. Full details of these models are provided in S2 Table and residual plots are shown in S1 Fig.

### Network size

The regression analysis for the number of incoming sharing ties shows that neither measure of household food access has any effect on network size: the posterior distributions of the coefficients for the predictors of incoming sharing ties (Fig 2a) indicate that neither lower harvest production nor wealth, as measured by the number of vehicles owned, are significantly associated with sharing network size. However, households that have more out-going sharing ties have more incoming ties.

Single-female-headed households have a slightly greater number of incoming ties than other household types. Importantly, single-female-headed households own fewer vehicles (Games-Howell test *p* = 0.007) and have lower harvest production levels (*χ*^2^ = 25.735, *p* < 0.001) than other households. The age of the eldest household member also has a positive effect on network size. However, it does not seem that older individuals have less access to country food through other means: the age of the oldest household member is actually mildly positively associated with the number of vehicles owned by the household (*r* = 0.248), and harvest production levels are not correlated with the age of the oldest household member (Games-Howell test yields no significant pairwise contrasts). This pattern likely reflects the fact that older Inuit often live in multigenerational households. Finally, households with more close kin may have slightly more incoming sharing ties, although the 95% posterior distribution interval for this variable includes zero (S2 Table).

### Network quality

Results for network quality (Fig 2b), measured by the number of sharing ties from high-harvest “super-households,” indicate that the strongest predictor of network quality is network size: households with more incoming sharing ties also have more ties from high-harvest households. After controlling for the effect of network size, neither poorer households nor households with lower harvests have a greater proportion of ties to high-production households than wealthier or higher harvest households. Network quality does not seem to vary much with age, but households headed by single women appear to have slightly lower network quality.

### Network density

Because network density is correlated with network size (larger networks tend to have lower density), the analysis of density must control for network size, as such, this analysis looks for trends in network density not related only to network size. The results (Fig 2c) show no significant effect on network density for any measure other than network size. Having a larger network is the only factor that determines whether a household has ties from more unconnected sources.

### Potential pathways to social capital

**Fig 3:**
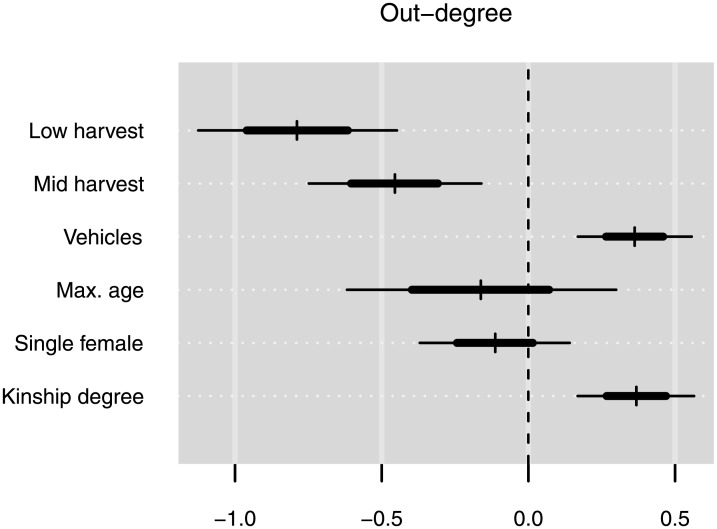
Posterior distributions of coefficients for selected predictor variables for sharing network out-degree. 68% and 95% quantiles of the distributions are shown by the thick and thin lines, respectively. The numeric variables, including the response variable, were log-transformed and centered. Low-harvest, mid-harvest, and single female are binary variables.

**Fig 4:**
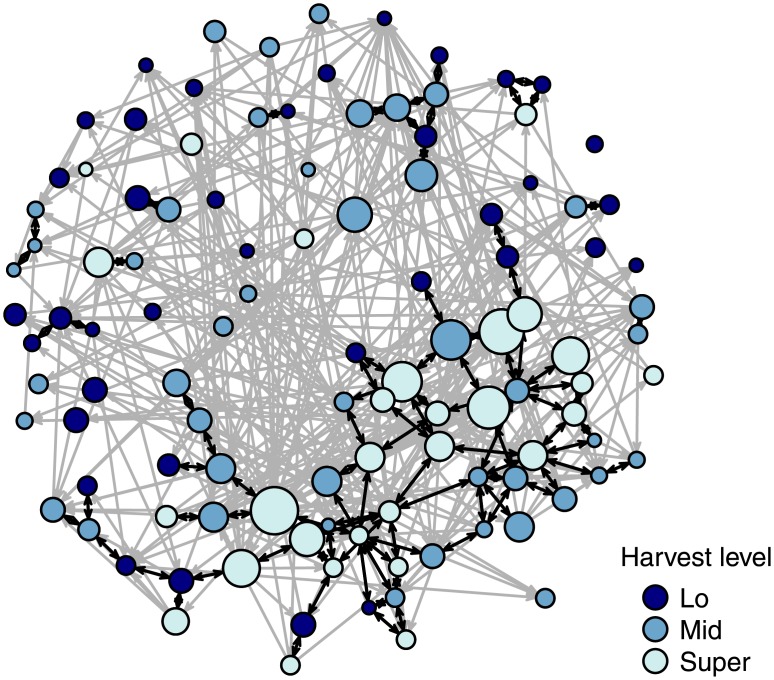
Country food sharing network showing the distribution of one-way (grey) and reciprocal (black) ties. Nodes are colored by the household’s harvest production level and sized based on the number of vehicles owned.

The three analyses presented above indicate that higher network quality and lower network density appear to be mainly associated with having a larger sharing network. Furthermore, the first analysis shows that, besides age, out-degree—giving away food—is the strongest predictor of network size. This suggests a link between generosity and social capital; that is, that incoming sharing ties might be induced by giving away country food. Further analyses comparing one-directional and mutual incoming ties indicate that the correlation between giving and receiving is driven entirely by dyadic reciprocity: households that give country food often receive country food back from the same households they give to (see S2 and S3 Figs and S3 Table). However, household wealth and harvest production are correlated with out-degree, as illustrated in Fig 3, which shows regression results for household out-degree (see S4 Table and S1 Fig for model details). Predictably, the results show that households with lower harvests have lower out-degree; while the number of vehicles owned (i.e., wealth) also has a significant positive effect on out-degree. In other words, because wealth and harvest production are positively correlated with giving food, they are indirectly associated with having more reciprocal sharing ties. Indeed, when reciprocal sharing ties are highlighted in the complete sharing network for the village, the associations between reciprocity, wealth, and harvest production levels of households are clearly visible (Fig 4). Interestingly, the results also show that although households with older persons receive from more other households, age itself is not a predictor of out-degree. That is, households with older persons do not give to more households than households with younger people—out-degree is instead only predicted by measures of wealth and harvest production. This is also true for kinship; households with more kin tend to have higher out-degree. However, households with more kin tend to be wealthier (i.e., own more vehicles) than households with fewer kin (*r* = 0.255).

## Discussion

In summary, households with more incoming sharing ties have less dense sharing networks and more numerous ties from super-households, and incoming sharing ties are in turn predicted by age, by being a single-female household-head, and by outgoing sharing ties. Perhaps surprisingly, the results indicate that neither household wealth nor harvest production are *directly* associated with the three measures of network social capital used here—either positively or negatively. What does this mean for the hypotheses proposed earlier?

First, the fact that households with low-wealth or low-harvest production do not have greater sharing-based social capital than more wealthy, higher-harvest production households means that the “trickle-down” hypothesis receives limited support: sharing ties in Kangiqsujuaq do not appear to be focused on households based on their need for food. It is important to emphasize that this result does not imply that current country food sharing does not produce a more equitable distribution of food resources among households in the settlement. Any food shares received by food insecure, low-harvest households certainly help them cope with food shortages. Further, the amount of country food shared between households was not available in this study, but flows of food may vary considerably between different ties, meaning that some households with few ties may still receive relatively large amounts of food. Nevertheless, in terms of network structures, households currently observed to have lower wealth and lower harvest production do not have more protective network structures surrounding them than more affluent, highly productive households.

Nevertheless, sharing ties in the network do reflect some cultural proxies for need. Age has a positive effect on sharing network size, as does being a single female household head. However, although single-female-headed households are more likely to be poor and to have low harvest production, the criteria of age, sex, and marital status are not perfectly aligned with actual food need. There are many low-income and/or low-harvest households that are headed or co-headed by young men, and these households are clearly not prioritized in the country food sharing network.

The higher in-degree of households with older persons should also be considered in light of other forms of sharing. In Inuit communities on Baffin and Victoria Islands, the households of elders often tend to serve as focal points for the collection and redistribution of country food within extended families [2, 9, 72]. Consequently, the increased number of ties of households with older persons in Kangiqsujuaq might reflect this kind of arrangement. A brief analysis of meal sharing network data collected for this project suggests that households with elders do provide meals to people from more other households than households with younger people do; but high harvest and wealthier households also host more others than households with fewer resources. Critically, members of food insecure households are not more likely to eat at more other people’s homes. That is, the preliminary analysis of meal sharing suggests that, although some elders’ households may serve as points of redistribution, consistent with the pattern in the country sharing network, sharing of meals is not biased towards people from food insecure households.

To summarize, country food sharing decisions are generally motivated by criteria other than household need (e.g., kinship and reciprocity), and when motivated by need, sharing is guided by traditional concepts of need and merit that do not directly map on to the realities of food insecurity in the contemporary economy. This bias partially reflects the fact that young men are expected to hunt for themselves [73], but households also have limited knowledge about broader patterns of food access in the settlement. This lack of information may partly explain the use of age, sex, and marital status as proxies for need. As one survey respondent noted:

“Maybe 10 years ago or more than 10, we would have no problem to have country food but there was a girl starving to death—we didn’t know which house didn’t have food. It was really a shock to us; now I always think of that and I wonder who needs food but I don’t know who.”

The increasing size of the village, as well as patterns of interaction among groups within the village (cf. [74]), mean that there are social barriers to sharing with those who need it despite a strong ethic of mutual aid.

In addition, the limits of sharing to serve as an effective redistributive mechanism are already being tested for some traditional resources in Nunavik. Both historic and ethnographic accounts indicate that severe levels of resource limitation may lead to hoarding, which exacerbates the divide between “haves” and “have-nots” when it comes to access to highly-desirable traditional foods [75–77]. For example, in Akulivik (on the Hudson Bay coast), after the imposition of a quota for beluga, hunters were observed to hide their catches in order to avoid sharing [78]. Because of the scarcity of beluga meat due to hunting quotas, similar behaviors sometimes occur in Kangiqsujuaq.

Clearly then, the “trickle-down” hypothesis, that sharing-based social capital might compensate for a lack of other resources in a household, receives only very limited, and strongly qualified, support. In contrast, sharing-based social capital does appear to be associated with households that give more, because of the connection between incoming and outgoing sharing ties that is driven by reciprocity within dyads. The analyses further show that higher wealth and harvest production increases a household’s ability to give, and households with more close kin in the village also have more expansive sharing networks. However, not all households have access to the resources, time, and knowledge required to sustain high levels of harvest production and food sharing. The combination of these two processes results in a broader pattern in the settlement, clearly visible in Fig 4, in which reciprocity occurs predominantly among highly productive hunters. In principle, this pattern supports the “insurance” hypothesis, which suggests that hunters may smooth out their access to country food through exchange with one another.

However, in the absence of data on quantities exchanged, the potential importance of food sharing as a form of insurance for hunters should not be over-emphasized. The availability of many resources, such as beluga, caribou, and geese, is highly seasonal and/or temporally limited; which means that the availability of many types of country food in the households of generalist hunters who are active year-round is often correlated. In addition, many high harvest households are also relatively wealthy. For both of these reasons, the insurance function of food sharing may be of limited economic importance to many high-harvest households. For these households, the importance of social bonds with other hunters, including sharing of information, is likely an important motivator for sharing in addition to any insurance benefit they receive.

Finally, country food sharing relationships in Kangiqsujuaq are sometimes associated with other flows of cash and supplies. Elsewhere I have suggested that such flows may be particularly associated with sharing from hunters to non-hunting but relatively affluent households [47]. The sharing-based social capital of some non-hunting households is likely enhanced by such relationships, and this pattern may account for some of the unexplained variance in the models presented here. However, the observation that poor, non-hunting households have limited social capital in the food sharing network (and limited means to build it), stands despite this possibility.

What might be the longer term consequences of the patterns of social capital observed here? Ecological resilience, “the persistence of systems and of their ability to absorb change and disturbance and still maintain the same relationships between populations or state variables” [79] (p. 14), has been widely adopted as a prescriptive concept in socio-ecological systems research, in the Arctic and globally [80–84]. In the Canadian Arctic, traditional food sharing has been suggested as one of the mechanisms that may promote community resilience [20, 25, 26, 85–88]. If sharing is included among adaptations that build resilience for communities and will assist them in coping with climate change, then it is essential to critically examine how, why, and under what circumstances sharing produces resilience. However, the question of how to measure resilience and sustainability in socio-ecological systems remains an open problem in climate change science [42, 89].

Clearly, to establish the response of sharing networks (food sharing and otherwise) to changes in resource availability, longitudinal studies will be required. However, the available data from Kangiqsujuaq suggest that larger, higher quality, and less redundant sharing network structures, which should enhance a household’s ability to access country food through sharing, are associated with generosity to others, with is in turn enabled by wealth. Giving patterns do not closely track food need but rather are driven by reciprocity and by traditional considerations that, in today’s economy, are neither exclusively nor always associated with poverty or food insecurity. This suggests that the country food access of households that are already poor and low harvest—especially households that are not single-female-headed or that do not comprise elders—may be more vulnerable to fluctuations in food availability than the networks of more wealthy households. This represents an important hypothesis to examine in future studies of the resilience of arctic food systems. While this study focuses on one form of social support in an Inuit settlement, the conclusion that reciprocity is highly important to promoting access to resources through social channels has broad theoretical relevance for understanding the role of social support and social capital in mitigating resource shocks in many socioeconomic systems. In particular, it highlights that even social institutions that appear to be focused on risk-management through mutual aid need to be carefully examined to accurately understand their long-term impacts on the well-being of the poor versus the wealthy [90]. Critically, the results suggest that socioeconomic factors (i.e., poverty and inequality) are extremely important in shaping household access to country food. Consequently, any analysis of socioecological resilience in arctic settlements should take into account how environmental, social and economic changes may interact with existing economic and social structures.

In conclusion, the results of this study clearly show that the notion that food sharing “maximizes all aspects of well-being in a community” [52] (p. 78) glosses over real differences within the settlement in both participation in sharing and in the rewards derived from it. Many households, due to a lack of adequate resources are unable to fully engage in harvesting and sharing [48], and these households consequently have limited ability to generate social capital through sharing. Given the patterns of household social capital documented in this research, sharing should not be considered as a palliative to climate change or other adaptive challenges facing northern settlements such as Kangiqsujuaq so long as high levels of poverty continue to undermine both store and country food access.

## Supporting information

S1 Table Description of the dataset. (PDF)

S2 Table Posterior distributions for models of network social capital measures. (PDF)

S3 Table Posterior distributions for regressions of one-way and mutual incoming sharing ties. (PDF)

S4 Table Posterior distributions for out-degree regression model. (PDF)

S1 Data Dataset used in the analysis. (CSV)

S1 Fig Mean residual plots for the models presented in the main text. (PDF)

S2 Fig Posterior distributions of coefficients for selected predictor variables for one-way and mutual incoming sharing ties. (PDF)

S3 Fig Mean residual plots for the models of one-way and reciprocal in-degree. (PDF)
