# Supplementary material for: Sharing-based social capital associated with harvest production and wealth in the Canadian Arctic
Source: PLoS One. 2018 Mar 12;13(3):e0193759. doi: 10.1371/journal.pone.0193759 (PMC5846769; doi:10.1371/journal.pone.0193759)
Supplement: S1 Table — (PDF) [file pone.0193759.s001.pdf]

S1 Table

Table 1: Description of the dataset provided in S1 Data.

Column name	Description
ID	Household ID number
Indegree	Number of households from which the household receives country food
Outdegree	Number of households to which the household gives country food
Superin	Number of super households from which the household receives country food
Mutual	Number of mutual (symmetric) ties each household has
Oneway	Number of one-way incoming ties each household has
Density	Density of the "in-neighborhood" of household's sharing networks. Ties from the focal household are not considered in the calculation
Huntcat	(Factor 0/1/2) Household harvest production level (low/mid/super). Households that did not harvest any of four key species (ringed seal, beluga, caribou, and geese) are "low production;" households in the top 30 percentile of production are considered "super-households"
Allvehicles	The number of vehicles owned by the household, including cars/trucks, snowmobiles, fishing boats and freighter canoes, and all-terrain vehicles
HHsize	Number of people living in the household. To protect the identity of respondents, this variable is provided after log-transformation and centering around the mean
Maxage	Age in years of the oldest member of the household. To protect the identity of respondents, this variable is provided after log-transformation and centering around the mean
Singf	(Factor 0/1) Whether the household is headed by a single woman
FMgive	(Factor 0/1) Whether the household gave food away over the local radio in the 12 months preceding the survey
Kinnum	Number of other households with siblings, parents, or children of HH members
