# Supplementary material for: Sharing-based social capital associated with harvest production and wealth in the Canadian Arctic
Source: PLoS One. 2018 Mar 12;13(3):e0193759. doi: 10.1371/journal.pone.0193759 (PMC5846769; doi:10.1371/journal.pone.0193759)
Supplement: S2 Table — (PDF) [file pone.0193759.s002.pdf]

S2 Table

Table 1: Posterior distributions for models of network social capital measures, with 2.5% and 97.5% quantiles

	Network size			Network quality			Network density		
Model term	Mean	2.5%	97.5%	Mean	2.5%	97.5%	Mean	2.5%	97.5%
Intercept	-0.098	-0.285	0.088	0.422	-0.115	0.966	0.015	-0.016	0.047
In-degree*	N/A	N/A	N/A	2.473	1.897	3.054	-0.139	-0.173	-0.106
Out-degree*	0.230	0.083	0.375	0.290	-0.158	0.740	-0.013	-0.039	0.013
Low production†	0.056	-0.226	0.337	-0.017	-0.831	0.805	-0.017	-0.064	0.030
Mid production†	-0.033	-0.262	0.202	-0.490	-1.164	0.182	-0.009	-0.048	0.029
Vehicles*	0.019	-0.135	0.171	-0.062	-0.508	0.380	0.000	-0.026	0.025
Household size*	0.146	-0.064	0.357	-0.096	-0.702	0.520	0.003	-0.033	0.039
Age oldest member*	0.929	0.584	1.272	0.598	-0.528	1.726	-0.044	-0.110	0.022
Single female headed†	0.279	0.082	0.476	-0.661	-1.243	-0.072	-0.018	-0.052	0.016
FM giving†	-0.051	-0.238	0.134	-0.047	-0.585	0.496	-0.008	-0.040	0.023
Close kin households*	0.141	-0.017	0.300	-0.333	-0.798	0.130	0.017	-0.010	0.044
τ	5.753	4.255	7.455	0.687	0.510	0.892	205.938	152.193	267.608

*denotes log-transformed variables, $log(x+1)$. Response variables were also log-transformed.

† denotes categorical (0/1) variables.
