# Supplementary material for: Sharing-based social capital associated with harvest production and wealth in the Canadian Arctic
Source: PLoS One. 2018 Mar 12;13(3):e0193759. doi: 10.1371/journal.pone.0193759 (PMC5846769; doi:10.1371/journal.pone.0193759)
Supplement: S3 Table — (PDF) [file pone.0193759.s003.pdf]

S3 Table

Table 1: Posterior distributions for regressions of one-way and mutual incoming sharing ties, with 2.5% and 97.5% quantiles

	One-way in-degree			Mutual ties		
Model term	Mean	2.5%	97.5%	Mean	2.5%	97.5%
Intercept	-0.163	-0.398	0.063	-0.001	-0.188	0.190
Out-degree*	-0.111	-0.293	0.074	0.598	0.450	0.746
Low production†	0.115	-0.230	0.471	-0.026	-0.316	0.261
Mid production†	0.087	-0.198	0.379	-0.092	-0.330	0.141
Vehicles*	0.066	-0.125	0.256	-0.037	-0.192	0.118
Household size*	0.142	-0.118	0.403	-0.045	-0.258	0.167
Age oldest member*	1.203	0.779	1.629	0.266	-0.081	0.616
Single female headed†	0.270	0.030	0.512	0.140	-0.054	0.338
FM giving†	-0.042	-0.275	0.189	-0.054	-0.244	0.137
Close kin households*	0.125	-0.071	0.320	0.070	-0.092	0.230
τ	3.757	2.774	4.866	5.637	4.175	7.303

*denotes log-transformed variables, $log(x+1)$. Response variables were also log-transformed.

† denotes categorical (0/1) variables.
