# Supplementary material for: Sharing-based social capital associated with harvest production and wealth in the Canadian Arctic
Source: PLoS One. 2018 Mar 12;13(3):e0193759. doi: 10.1371/journal.pone.0193759 (PMC5846769; doi:10.1371/journal.pone.0193759)
Supplement: S4 Table — (PDF) [file pone.0193759.s004.pdf]

S4 Table

Table 1: Posterior distributions for out-degree regression model, with 2.5% and 97.5% quantiles

	Out-degree		
Model term	Mean	2.5%	97.5%
Intercept	0.482	0.256	0.708
Low production †	-0.789	-1.125	-0.448
Mid production †	-0.456	-0.749	-0.160
Vehicles*	0.363	0.169	0.557
Household size*	0.016	-0.266	0.298
Age oldest member*	-0.163	-0.618	0.300
Single female headed †	-0.114	-0.370	0.141
FM giving †	0.050	-0.202	0.302
Close kin households*	0.368	0.168	0.565
τ	3.196	2.374	4.134

*denotes log-transformed variables, $log(x + 1)$. The response variable was also log-transformed.
† denotes categorical (0/1) variables.
