# Supplementary figures and images for: Sharing-based social capital associated with harvest production and wealth in the Canadian Arctic
Source: PLoS One. 2018 Mar 12;13(3):e0193759. doi: 10.1371/journal.pone.0193759 (PMC5846769; doi:10.1371/journal.pone.0193759)

## S1 Figure

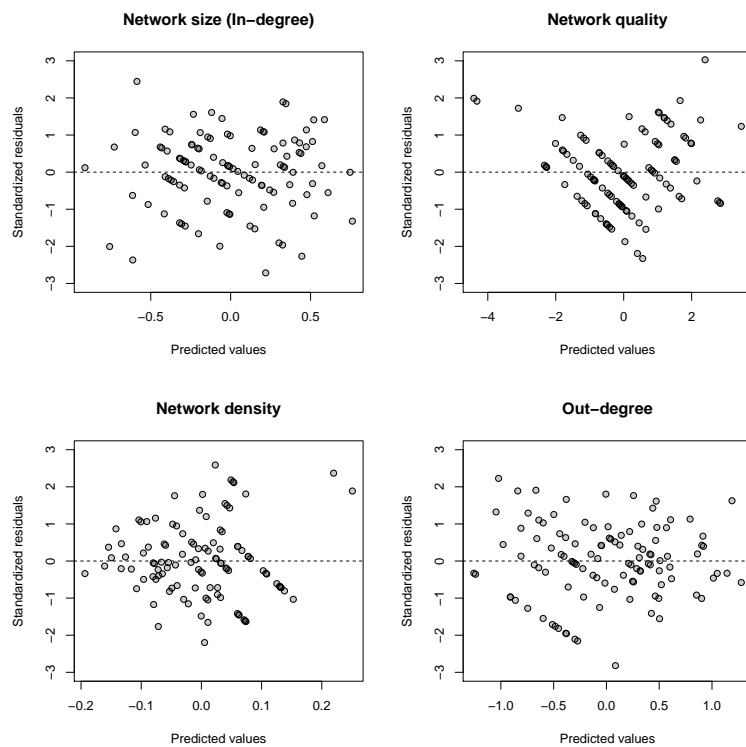

Figure 1: Mean residual plots for the models presented in the main text.

Supplement: S1 Fig — (PDF) [file pone.0193759.s006.pdf]

### S3 Figure

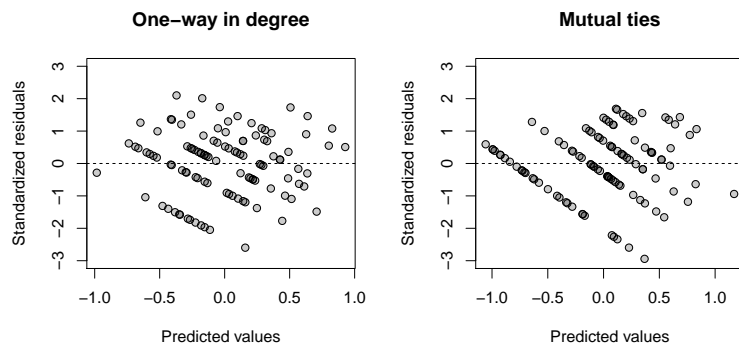

Figure 1: Mean residual plots for the models of one-way and reciprocal in-degree.

Supplement: S3 Fig — (PDF) [file pone.0193759.s008.pdf]
