# Supplementary material for: Sharing-based social capital associated with harvest production and wealth in the Canadian Arctic
Source: PLoS One. 2018 Mar 12;13(3):e0193759. doi: 10.1371/journal.pone.0193759 (PMC5846769; doi:10.1371/journal.pone.0193759)
Supplement: S2 Fig — (PDF) [file pone.0193759.s007.pdf]

S2 Figure

Additional regressions that divide the sharing network into two kinds of ties: one-way and reciprocal. In the latter case, the household receives food from another household that they also give back to. Figure 1 clearly shows that the relationship between sharing and out-degree is driven by mutual, not one-directional, ties. Thus, reciprocity in food sharing in Kangiqsujuaq appears to be contingent rather than generalized. The models were run using the settings described in the Data and Methods section. The posterior distributions for the models are described in S3 Table and residual plots are provided in S3 Fig.

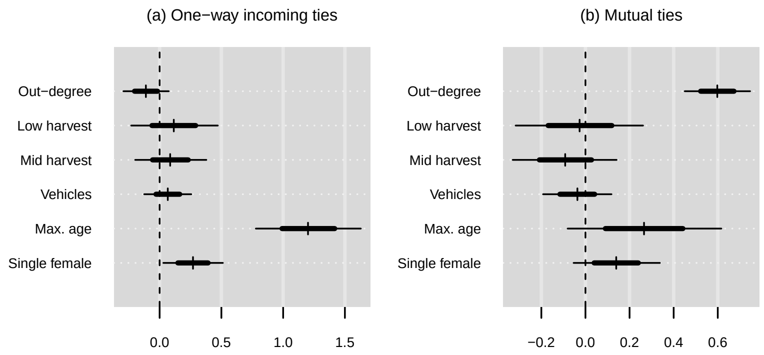

Figure 1: Posterior distributions of coefficients for selected predictor variables for one-way and mutual incoming sharing ties. 68% and 95% quantiles of the distributions are shown by the thick and thin lines, respectively. The numeric variables, including the response variable, were log-transformed and centered. Low-harvest, mid-harvest, and single female are binary variables.
